# *Lactobacillus acidophilus* VB1 co-aggregates and inhibits biofilm formation of chronic otitis media-associated pathogens

**DOI:** 10.1007/s42770-024-01363-5

**Published:** 2024-05-24

**Authors:** Ammar R. Algburi, Shireen M. Jassim, Igor V. Popov, Richard Weeks, Michael L. Chikindas

**Affiliations:** 1 Department of Microbiology, Veterinary Medicine College, University of Diyala, Baqubah, Iraq; 2 Alkhalis Section for Primary Care/Thoracic and Respiratory Diseases Unit, Alkhalis, Iraq; 3 Centre for Healthy Eating and Food Innovation, Maastricht University-Campus Venlo, Venlo, The Netherlands; 4 Agrobiotechnology Center and Faculty “Bioengineering and Veterinary Medicine”, Don State Technical University, Rostov-On-Don, Russia; 5 Division of Immunobiology and Biomedicine, Center of Genetics and Life Sciences, Federal Territory Sirius, Sirius University of Science and Technology, Sochi, Russian Federation; 6 Health Promoting Naturals Laboratory, School of Environmental and Biological Sciences, Rutgers State University, New Brunswick, NJ USA; 7 I.M. Sechenov First Moscow State Medical University, Moscow, Russia

**Keywords:** Anti-biofilm activity, Antimicrobial combinations, Chronic otitis media, Ciprofloxacin, *Lactobacillus acidophilus* VB1

## Abstract

This study aims to evaluate the antibacterial activity of *Lactobacillus acidophilus*, alone and in combination with ciprofloxacin, against otitis media-associated bacteria. *L. acidophilus* cells were isolated from Vitalactic B (VB), a commercially available probiotic product containing two lactobacilli species, *L. acidophilus* and *Lactiplantibacillus* (formerly *Lactobacillus) plantarum*. The pathogenic bacterial samples were provided by Al-Shams Medical Laboratory (Baqubah, Iraq). Bacterial identification and antibiotic susceptibility testing for 16 antibiotics were performed using the VITEK2 system. The minimum inhibitory concentration of ciprofloxacin was also determined. The antimicrobial activity of *L. acidophilus* VB1 cell-free supernatant (La-CFS) was evaluated alone and in combination with ciprofloxacin using a checkerboard assay. Our data showed significant differences in the synergistic activity when La-CFS was combined with ciprofloxacin, in comparison to the use of each compound alone, against *Pseudomonas aeruginosa* SM17 and *Proteus mirabilis* SM42. However, an antagonistic effect was observed for the combination against *Staphylococcus aureus* SM23 and *Klebsiella pneumoniae* SM9. *L. acidophilus* VB1 was shown to significantly co-aggregate with the pathogenic bacteria, and the highest co-aggregation percentage was observed after 24 h of incubation. The anti-biofilm activities of CFS and biosurfactant (BS) of *L. acidophilus* VB1 were evaluated, and we found that the minimum biofilm inhibitory concentration that inhibits 50% of bacterial biofilm (MBIC50) of La*-*CFS was significantly lower than MBIC50 of La-BS against the tested pathogenic bacterial species. *Lactobacillus acidophilus*, isolated from Vitane Vitalactic B capsules, demonstrated promising antibacterial and anti-biofilm activities against otitis media pathogens, highlighting its potential as an effective complementary/alternative therapeutic strategy to control bacterial ear infections.

**Supplementary Information:**

The online version contains supplementary material available at 10.1007/s42770-024-01363-5.

## Introduction

Otitis media (OM) is a middle ear infection in adults and children, most commonly caused by multidrug-resistant bacteria and associated with various complications, including hearing loss, persistent ear effusion, mastoiditis, and chronic otitis media [1, 2]. OM describes several related clinical conditions including acute OM (AOM), chronic OM (COM), and COM with effusion (COME), also referred to as nonsuppurative OM. Chronic suppurative otitis media (CSOM) is a persistent or recurrent otorrhoea noticed after 2 to 6 weeks of bacterial attack due to either a rupture of the tympanic membrane or a ventilation tube and has also been defined as chronic active mucosal otitis media, chronic otomastoiditis, or chronic tympanomastoiditis [2]. CSOM is most common in children and most often occurs as a sequelae of acute otitis media [3]. The incidence of CSOM is estimated at more than 20 million people worldwide, with roughly 80% of preschoolers having experienced at least one acute otitis media (AOM) infection before their third birthday and almost 40% having had six or more recurring infections by age seven [4].

The public health impact of CSOM varies significantly between countries based on factors such as suppurative complications prevalence like mastoiditis, meningitis incidence rate, and sequelae development likelihood associated with CSOM resulting in hearing loss [5]. CSOM can have profound health implications and significant morbidity among those affected. It can lead to irreversible complications such as persistent otorrhea, mastoiditis, labyrinthitis, and facial palsy, as well as more severe complications, including intracranial abscesses and thromboses [5]. The potential for adverse outcomes and high incidence rates make CSOM a significant public health issue that deserves immediate attention.

A variety of different microbes (bacteria, fungi, viruses) can play a role in acute and/or chronic otitis media infections [6]. Common bacterial pathogens associated with OM and recurrent OM include *Haemophilus influenzae, Streptococcus pneumoniae*, and *Streptococcus pyogenes* [7, 8], and several studies have reported that the most isolated bacterial agents of CSOM are *Pseudomonas aeruginosa, Staphylococcus aureus, Staphylococcus epidermidis*, and *Klebsiella* spp [9, 10].. Attachment to and colonization of the middle ear by the most pathogenic bacteria associated with OM will lead to biofilm formation [11].

A biofilm is a bacterial community of aggregated cells that attach to a suitable surface and become protected from exterior stressors by a matrix of excreted polymeric substances composed mainly of proteins, polysaccharides, and extracellular DNA [12]. Biofilm formation is essential for most pathogenic bacteria to avoid host immune mechanisms, survive in harsh conditions, tolerate high concentrations of antibiotics, and establish a persistent infection [13].

In the United States, AOM is the most common reason for prescribing antibiotics to children, with the number of AOM patients treated with antibiotics reaching levels as high as 86–91% in the US in a study covering the 2011–2016 period [14]. Ciprofloxacin is commonly prescribed by Ear, Nose, and Throat (ENT) physicians to control bacterial OM; however, bacterial resistance to the antibiotic has been reported [15, 16]. Another analysis revealed that the rate of antibiotic-resistant ear infections in northeastern Ethiopia doubled from 2001 to 2011. Ampicillin had the highest overall resistance rate (88.5%), followed by ceftriaxone (84.5%), amoxicillin (81.9%), and tetracycline (74.5%) [17]. A rise in antimicrobial resistance for *P. aeruginosa* and *Haemophilus influenzae* isolates in the UK also appears to have increased from 2013 to 2018 [18]. The high frequency of antibiotic use for OM patients coupled with rising rates of antibiotic resistance in clinically relevant OM pathogens is cause for alarm. Therefore, there is an urgent need to investigate alternative antimicrobial substances with low resistance development potential and the ability to control biofilm formation in persistent infections, particularly those associated with drug-resistant pathogens.

Probiotics are described by the World Health Organization (WHO) as “live microorganisms that, when administered in adequate amounts, confer a health benefit on the host” which is cited by Binda et al. [19]. The antimicrobial activities of lactobacilli against a wide range of bacterial pathogens have been reported in many publications [20]. Some studies have demonstrated that lactobacilli produce broad-spectrum antimicrobials such as bacteriocins and anti-adherence biosurfactant molecules that are effective in controlling Gram-positive and Gram-negative pathogens [21, 22]. In addition, lactobacilli can suppress virulence factors and prevent the spread of pathogenic bacteria [23] by producing organic acids and other antimicrobial agents [24, 25]. It was reported that probiotic lactobacilli prevented the growth and biofilm formation of *Streptococcus mutans*, for example [26]. The anti-biofilm potential of probiotic lactobacilli is an effective tool for combating and preventing various bacterial infections, including oral, enteric, and urogenital pathogens [27, 28]. Moreover, probiotic lactobacilli play a crucial role in modulating the gut microbiota, enhancing host immune system functions, and producing inhibitory substances to inhibit biofilm formation [29].

Our study assesses the antimicrobial and anti-biofilm effect of *L. acidophilus* VB1 and its metabolites against CSOM-associated bacteria. It also investigates the potential synergy of antimicrobial combinations of ciprofloxacin with the cell-free supernatant (CFS) of the *Lactobacillus* species against the selected pathogens.

## Materials and methods

### Growth conditions, isolation, and identification of bacterial isolates

This study included the most common bacterial species isolated from patients with CSOM in the ENT section of the Baquba Teaching Hospital, Diyala, Iraq. The bacterial isolates were identified as *P. aeruginosa* SM17, *S. aureus* SM23, *P*. *mirabilis* SM42, and *K*. *pneumoniae* SM9. The confirmation of bacterial species identification and their antimicrobial susceptibility 16 antibiotics were performed using the VITEK 2 system (BioMérieux, Marcy-l’Étoile, France). The VITEK 2 system was also used to assess the antibiotic susceptibility of the bacterial isolates using an AST-GN card for Gram-negative and an AST-GP card for Gram-positive bacteria. *L. acidophilus* VB1 was isolated from the Vitalactic B dual-species probiotic supplement containing a mixed culture of the probiotic preparation, *L. plantarum*, and *L. acidophilus*, using a pure culture technique, and identification was confirmed using the VITEK2 system. Probiotic products were purchased from Vitane Pharmaceuticals, Inc. (Congers, NY, USA) and maintained in skimmed milk for 48 h at 37 °C under aerobic conditions (please see supplementary Table S1). After incubation, 10 µl of the lactobacilli cell suspensions were streaked onto De Man, Rogosa, and Sharpe (MRS) agar (Liofilchem, Roseto degli Abruzzi, Italy) and incubated under the same above-mentioned conditions.

### Antibiogram assay of the isolated pathogens and the *L. acidophilus* VB1

The Kirby-Bauer method was used following Clinical and Laboratory Standards Institute (CLSI) methods [30] to evaluate antibiotic susceptibility to ciprofloxacin, which was selected based on the recommendation of WHO [31].

### Preparation of La-CFS and La-BS

The La-CFS was prepared according to Pompilio et al. [32] with minor modifications. *L. acidophilus* VB1 was inoculated into MRS broth and incubated at 37 °C for 24 h under aerobic conditions. The bacterial cells were centrifuged using 4800 *g* at 4 °C for 30 min, and the CFS was then collected. The CFS was filtered using a sterilized syringe filter, Millipore 0.45 μm (Difco Laboratories, Franklin Lakes, NJ, USA) and kept at 4 °C.

The biosurfactant was isolated from the *L. acidophilus* VB1 following the methods of Sambanthamoorthy et al. [33] with minor modifications. Briefly, 300 ml of MRS broth was inoculated with 5 ml of the overnight culture of *L. acidophilus* VB1 and then incubated overnight at 37 °C under aerobic conditions. After incubation, the cells were precipitated by centrifugation using 8500 *g*, at 10 °C, for 10 min, washed twice in distilled water, and re-suspended in 100 ml of phosphate-buffered saline (PBS). The suspension was placed into a shaker incubator for 2 h at room temperature to release the lactobacilli-bound BS. Then, the bacterial cells were separated by centrifugation, and the supernatants were collected and filtered using a 0.45 μm syringe filter. The sterile supernatant, a stock solution of La-BS, was kept at 4 °C for further use.

### Co-aggregation test

The co-aggregation assay was adapted from the method of Algburi et al. [34]. Briefly, the isolated bacteria and *L. acidophilus* VB1 were cultured separately and incubated aerobically at 37 °C for 24 h in brain heart infusion (BHI) and MRS broth, respectively. The planktonic cells were harvested by centrifugation using 4480 *g*, 15 min, 23 °C. Cells were washed twice and re-suspended in PBS, and their optical density OD_630 nm_ was adjusted to 0.25. A 100 µl suspension of each bacterial dilution, lactobacilli, and pathogenic bacteria was mixed into a 96-well microplate to a final volume of 200 µl. A 200 µl suspension of each bacterial dilution was added to the microplate separately to evaluate bacterial auto-aggregation. The plates were incubated at 37 °C without shaking, and OD_630 nm_ was recorded at 0, 2, 4, and 24 h. The calculation of the co-aggregation percentage was based on the equation below [35]:

$$ \text{C}\text{o} - \text{a}\text{g}\text{g}\text{r}\text{e}\text{g}\text{a}\text{t}\text{i}\text{o}\text{n} \text{\%} = \frac{(\text{X} - \text{Y})}{\left(X\right)} \times 100$$

The (X) refers to the absorbance before incubation at time 0, and Y indicates the absorbance after incubation at a given time point at time 2, 4, and 24 h. The assay was performed in duplicates.

### Determination of minimum inhibitory concentration (MIC)

The MICs of La*-*CFS that inhibit 90% of the growth of the tested isolates, the MIC90, were determined differently against CSOM pathogenic bacteria according to AL-Dulaimi et al. [36], with minor modifications. Briefly, La*-*CFS 100% was prepared as a stock solution and then two-fold diluted into 96 wells of the microplate with 75 µl of fresh BHI broth. Then, each well was inoculated individually with 75 µl of 1.5 × 10^8^ CFU ml^− 1^ of the bacterial suspension to a final volume of 150 µl in each well. The positive (untreated bacterial cells) and the negative (broth only, antimicrobial diluted in BHI) controls were included in this assay. The microplates were incubated aerobically at 37°C for 24 h. Then, the MIC90 was determined using a microplate reader (Molecular Diagnostics, Sunnyvale, CA, USA) at OD_630 nm_. The MIC90 was defined as ‘’the lowest concentration of antimicrobial that can inhibit 90% of bacterial growth compared to the positive control after 24 hours incubation’’ [37].

### Checkerboard assay for antimicrobial combinations

A checkerboard assay was used to investigate the efficacy of antimicrobial combinations between La*-*CFS and ciprofloxacin against the selected CSOM bacterial pathogens following Bellio et al. [38] with minor modifications. Briefly, the antimicrobials were serially diluted two-fold into fresh BHI broth using two separate 96-well microplates. A 50 µl aliquot from each dilution of antimicrobial A (La*-*CFS) was transferred horizontally to 50 µl of antimicrobial B (ciprofloxacin). Then, 100 µl of bacterial suspension 1.5 × 10^8^ CFU ml^− 1^ was added to the antimicrobial combinations (A & B) in each well. Ciprofloxacin 15.6–0.24 µg ml^− 1^ and La*-*CFS 50-6.25% concentrations were prepared according to their previously determined MICs. Also, positive and negative controls were used in duplicate. After 24 h incubation, the MIC value for each CFS and antibiotic, alone and in combination, were identified using a microplate reader at OD_630 nm_. Isobolograms were used to compare the MIC values and determine whether antimicrobial mixtures are synergized or antagonized against the tested pathogenic bacteria. Our data were analyzed and described as previously indicated by Turovskiy & Chikindas [39].

### Anti-biofilm activity of La-CFS and La-BS

The MBIC50 of *L. acidophilus* VB1metabolites (La-CFS and La-BS) were identified as described by Algburi et al. [40], with minor modifications. Briefly, La-CFS and La-BS were diluted two-fold with an appropriate volume of fresh BHI broth supplemented with 1% glucose (BHIG) in a 96-well microplate. Bacterial cells, 1.5 × 10^8^ CFU ml^− 1^, were separately transferred into each well containing pre-determined concentrations of *Lactobacillus* CFS/BS. Positive (BHIG broth inoculated with bacterial cells) and negative controls (BHIG broth only) were included in this assay. The microplates were covered with a lid and incubated at 37 °C under aerobic conditions for 24 h. Following incubation, non-adherent cells were removed from each well without disrupting the biofilm and transferred into new microplates, and their turbidity was measured at OD_630_. The wells were then gently washed three times with 200 µl of distilled water. The biofilm cells were fixed by heating for 60 min at 60 °C in the oven. After fixation, 100 µl of 0.1% crystal violet (CV) (BDH, Lutterworth, England) was added to each treated well and left for 15–20 min at room temperature. The residue of crystal violet was removed, and each well was washed three times with 200 µl of distilled water and air-dried. Then, 150 µl of 95% ethanol was added to each well to solubilize the dye bound to the adherent cells. The microplates were then incubated for 30 min at 4 °C. After incubation, 125 µl of the solubilized CV were transferred from each treated well into new 96 well microplates, and absorbance at OD_630_ was determined. The biofilm inhibition percentages were then calculated compared to the positive control.

### Statistical analysis

Sigma plot V.11 (Informer Technologies Inc, Roseau, DOMINICA**)** and SPSS V.20 (IBMSPSS®, Armonk, New York, USA) software were used to analyze continuous variables, and the mean and standard error were calculated. The data had normal distribution according to the Shapiro-Wilk test. Statistical analysis was performed using the one-way analysis of variance (ANOVA) test. followed by Tukey’s test. The programming language R v4.2.3 (R Foundation for Statistical Computing, Vienna, Austria) and the “ggplot2” package were used for the data visualization. *P* ≤ 0.05 indicates a statistically significant difference between measured values.

## Results

### Bacterial identification and antibiotic susceptibility testing of bacterial isolates

The VITEK 2 system found that the bacterial isolates were 95–99% identified as *P. aeruginosa*, *S. aureus*, *P*. *mirabilis, K*. *pneumoniae* and designated as SM17, SM23, SM9, and SM42, respectively (please see Table S1). Based on the VITEK 2 system data, we also found that all bacterial isolates were resistant to piperacillin, gentamicin, and tobramycin. In addition, *P. aeruginosa* SM17 and *K. pneumoniae* SM9 were both resistant to ceftazidime and aztreonam. Both *K. pneumoniae* SM9 and *P. mirabilis* SM42 were also resistant to ticarcillin, ticarcillin/clavulanic, and trimethoprim/sulfamethoxazole. The *S. aureus* SM23 isolate was resistant to benzylpenicillin and oxacillin.

### Minimum inhibitory concentrations of La-CFS and ciprofloxacin

In this work, the broth microdilution method was used to determine MIC90 values. In this assay, A series of two-fold dilutions were prepared from 500–0.95 µg ml^− 1^ for ciprofloxacin and from 50 to 6.25% for La-CFS. The MIC90 of ciprofloxacin was 0.95–1.9 µg ml^− 1^, while those of the CFS were in the range of 25–50% against CSOM-isolated bacteria.

**Fig. 1:**
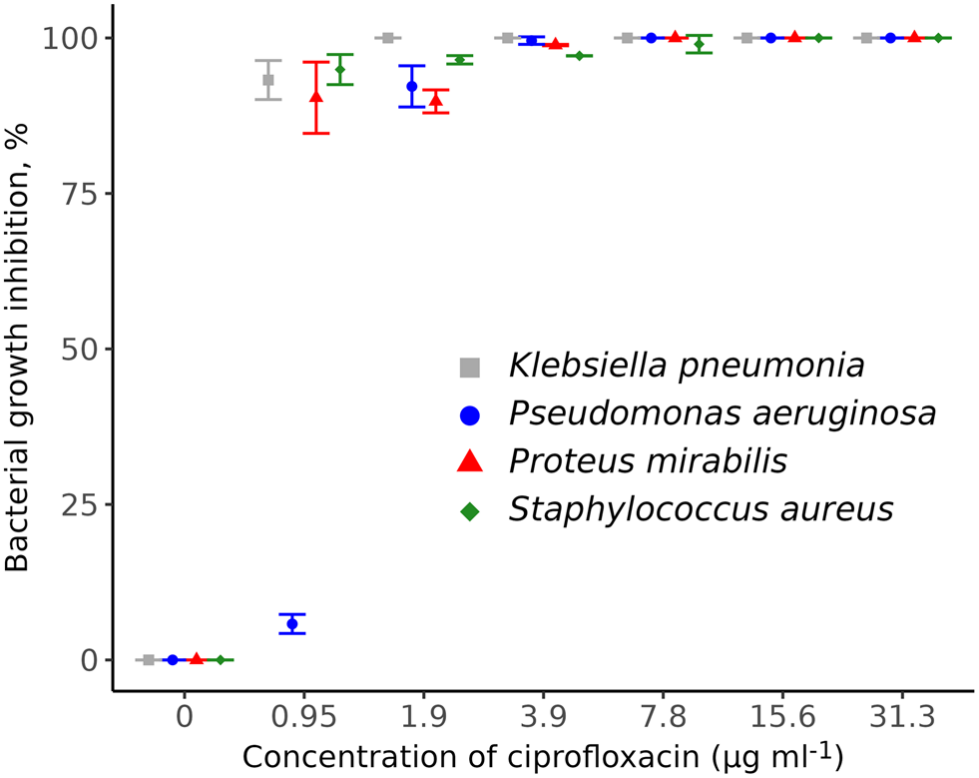
Antibacterial activity of ciprofloxacin against bacterial isolates

In comparison to the control, *P. aeruginosa* SM17 displayed a significant inhibition of 92.1% (*P* < 0.001) when 1.9 µg ml^− 1^ of ciprofloxacin was applied (Fig. 1). The MIC90 value was 0.95 µg ml^− 1^ for *P. mirabilis* SM42, *K*. *pneumoniae* SM9, and *S. aureus* SM23 with recorded growth inhibition of 90.3%, 93.3%, and 94.9%, respectively. The results showed that the bacterial growth inhibition percentages were significantly different (*P* < 0.001) when the tested concentrations of ciprofloxacin (31.3, 15.6, 7.8, 3.9, 1.9, and 0.95) µg ml^− 1^ were used as compared to the positive control (Table S2).

**Fig. 2:**
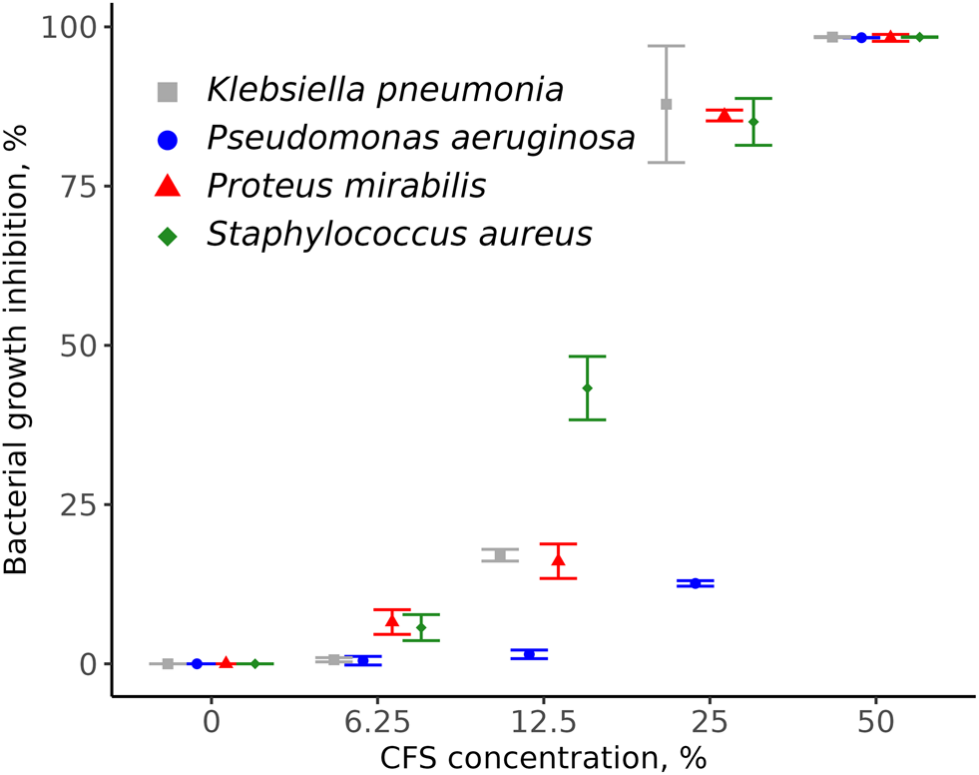
Antibacterial activity of La-CFS against bacterial isolates

The MIC90 of La-CFS was 50% against *P. aeruginosa* SM17 and inhibited 98.3% of bacterial growth (Fig. 2). In addition, 25% La-CFS prevented 92.3%, 89.6%, and 90.01% of *K. pneumoniae* SM9, *P. mirabilis* SM42, and *S. aureus* SM23 growth, respectively, as compared to the control. With the exception of 6.25%, a significant difference in bacterial growth inhibition was reported when 12.5%, 25%, and 50% La-CFS were used compared to the control (*P* < 0.05; Table S3).

### The activity of La-CFS in combination with ciprofloxacin against bacterial isolates

**Fig. 3:**
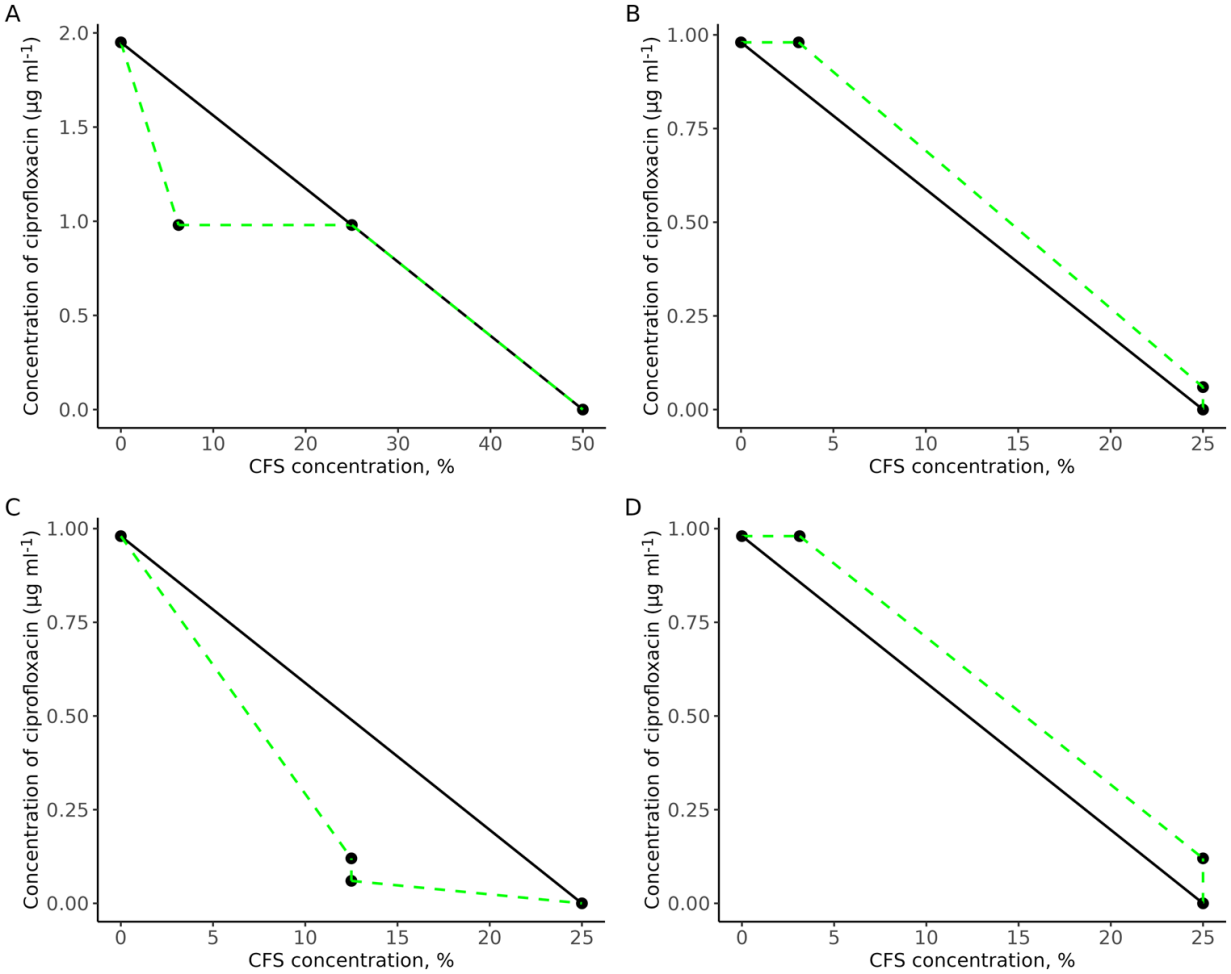
Antimicrobial combinations of ciprofloxacin with La-CFS against the tested bacterial species. **(A)** Synergism effect against *P. aeruginosa* SM17. **(B)** Antagonism effect against *S. aureus* SM23. **(C)** Synergism effect against *P. mirabilis* SM42. **(D)** Antagonism effect against *K. pneumoniae* SM9

A checkerboard assay and isobolograms were used to evaluate the potential synergy of the antimicrobial combinations of ciprofloxacin and La-CFS against the bacterial isolates. A synergistic effect was observed when La-CFS was combined with ciprofloxacin against *P. aeruginosa* SM17. When combined, the MICs were 0.98 µg ml^− 1^ for ciprofloxacin and 6.25% and 25% for La-CFS compared to ciprofloxacin (1.9 µg ml^− 1^) and La-CFS (50%) MICs when used alone (Fig. 3A). Ciprofloxacin was antagonized in combination with La-CFS against *S. aureus* SM23, the MICs of ciprofloxacin were 0.98 µg ml^− 1^ and 0.07 µg ml^− 1^ when combined with 3.31% and 25% of La-CFS, in comparison to the MICs of ciprofloxacin (0.95 µg ml^− 1^) and CFS (25%) when used alone (Fig. 3B). A synergistic effect was reported when ciprofloxacin was combined with La-CFS against *P. mirabilis* SM42; the MICs of ciprofloxacin were 0.13 and 0.07 µg ml^− 1^ when combined with 12.5% of La-CFS, compared to the MICs of ciprofloxacin (0.95 µg ml^− 1^) and La-CFS (25%) alone (Fig. 3C). An antagonistic effect was observed when La-CFS was mixed with ciprofloxacin against *K. pneumoniae* SM9; the ciprofloxacin MICs were 0.98 µg ml^− 1^ and 0.13 µg ml^− 1^ when combined with 3.31% and 25% of La-CFS (Fig. 3D).

### *Lactobacillus acidophilus* VB1 co-aggregates with the isolated pathogens

**Fig. 4:**
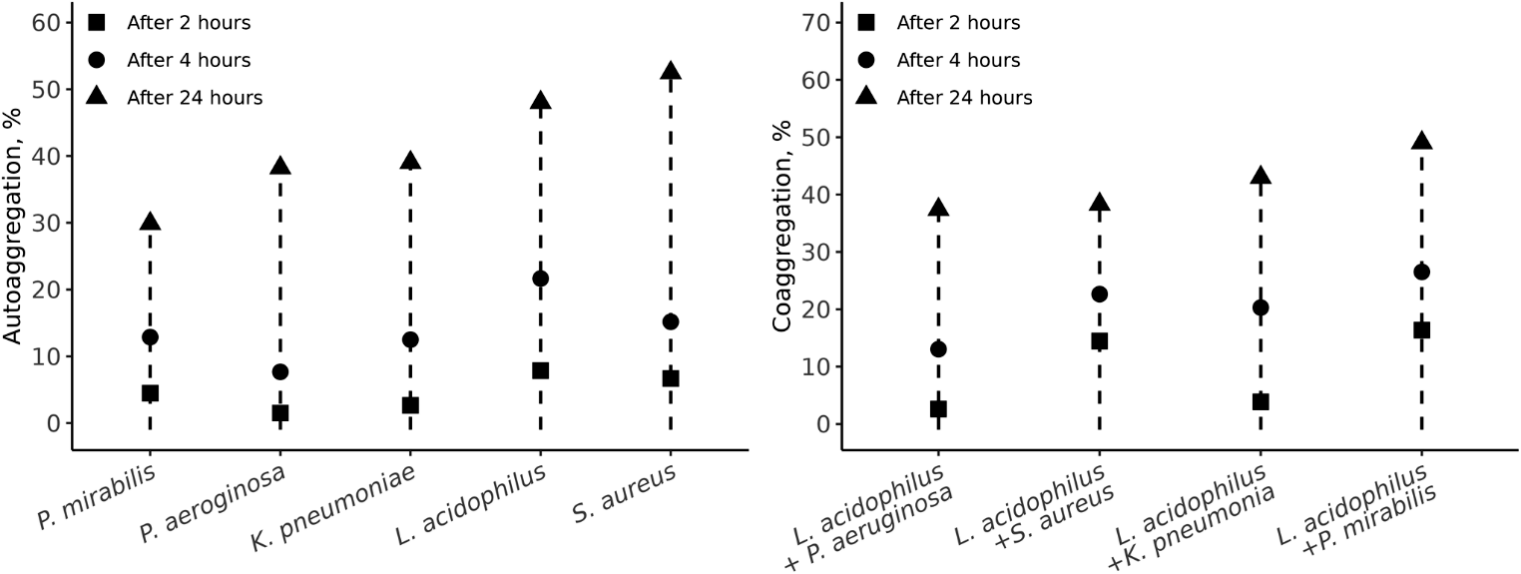
The auto-aggregation and co-aggregation %. **(A)** auto-aggregation % of the selected bacterial species. **(B)** The co-aggregation % between the tested bacterial isolates

The coaggregation of lactobacilli with CSOM bacterial isolates was evaluated using an automated microtiter plate reader at a wavelength of 630 nm at 0, 2, 4, and 24 h time points. The highest auto-aggregation percentages after 24 h of incubation were 52.5% for *S. aureus* SM23, followed by *L. acidophilus* VB1, *K. pneumoniae* SM9, *P. mirabilis* SM42, and *P. aeruginosa* SM17, which were 48.02%, 39.02%, 38.2%, and 29.9%, respectively (Fig. 4A).

Similarly, the highest coaggregation percentages (49.04%) were reported when *L*. *acidophilus* VB1, after 24 h, was mixed with *P*. *mirabilis* SM42. When *L. acidophilus* was added to, *K. pneumoniae* SM9, *S. aureus* SM2,3, *and P. aeruginosa* SM17, the percentages of coaggregation were 42.9%, 38.3%, and 37.4%, respectively (Fig. 4B).

### Anti-biofilm activity of La-CFS and La-BS

In this study, we determined the MBIC50 of both La-CFS and La-BS, which is defined as the lowest concentration of an antimicrobial that inhibits ≥ 50% of biofilm formation compared to an untreated control (bacterial biofilm without antimicrobial treatment).

**Fig. 5:**
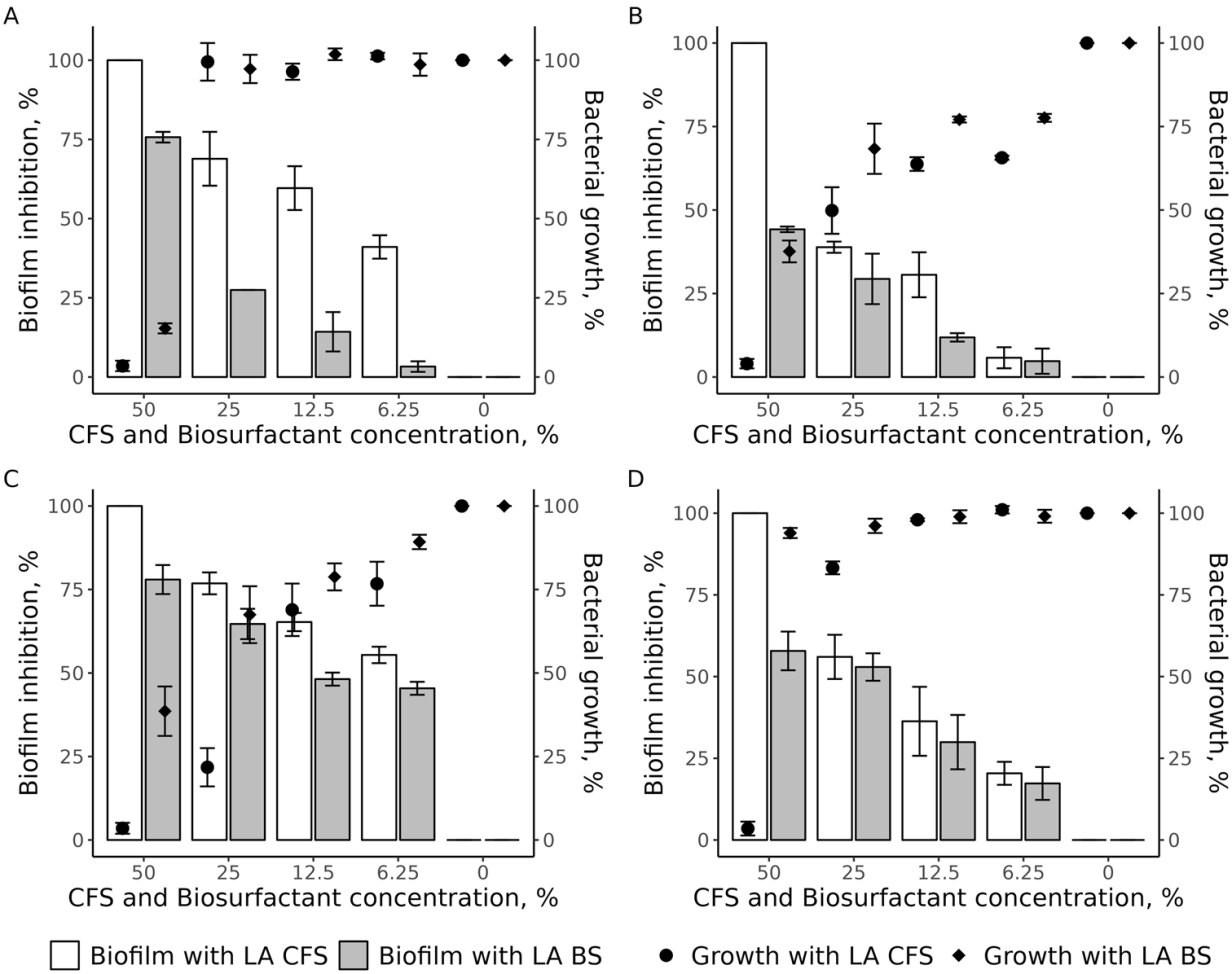
The effect of CFS and BS of *L. acidophilus* VB1 on the biofilm and planktonic growth of the tested bacterial species. Results expressed as mean MBIC50±SD (%) to three independent experiments. **(A)** Effect of CFS and BS of *L. acidophilus* VB1 on *P. aeruginosa* SM17. **(B)** Effect of CFS and BS of *L. acidophilus* VB1 on *S. aureus* SM23. **(C)** Effect of CFS and BS of *L. acidophilus* VB1 on *P. mirabilis* SM42. **(D)** Effect of CFS and BS of *L. acidophilus* VB1 on *K. pneumoniae* SM9

Our data showed that the MBIC50 of La-CFS was 12.5%, inhibiting 59.7% of *P. aeruginosa* SM17 biofilm, while the La-BS MBIC50 was 50%, inhibiting 75.6% of bacterial biofilm (Fig. 5A). Significant differences were identified in biofilm formation inhibition between La-CFS and La-BS at all tested concentrations compared to the control (*P* < 0.05). In the same regard, there were significant differences in the planktonic growth percentages when 50% of both La-BS and La-CFS were used against *P. aeruginosa* SM17, (*P* < 0.05; Table S4A).

We observed that 100% of *S. aureus* SM23 biofilm was prevented when 50% La-CFS was applied. While using 50% La-BS reduced only 44.6% of bacterial biofilm (Fig. 5B). Significant differences were also observed in biofilm formation inhibition when 50% of La-CFS and La-BS were used (*P* < 0.05; Table S4B). Regarding bacterial growth, a significant reduction (*P* < 0.001) was reported, compared to the control, when 50%, 12.5%, and 6.25% of La-CFS and La-BS were applied (Table S4C).

Significant differences were observed in biofilm inhibition between La-CFS and La-BS at all tested concentrations, except 25%, (Table S4C). For P. mirabilis SM42, a significant reduction of 54.9% of biofilm was observed at MBIC50 (6.25%; *P* < 0.05), Whereas the MBIC50 of La-BS (25%) inhibited 64.6% of biofilm formation (Fig. 5C). Similarly, significant differences (*P* < 0.05) in bacterial cell viability were reported when 50% and 25% La-CFS and La-BS were used (Table S4C).

Figure 5D shows that *K. pneumoniae* SM9 biofilm inhibition was 56.1% when 25% (as an MBIC50) of La-CFS was used, while the MBIC50 of La-BS was 50% with 58.6% of *K. pneumoniae* SM9 biofilm being inhibited. Significant differences were identified in biofilm formation inhibition between using 50% of La-CFS and La-BS (*P* < 0.05; Table S4D). Regarding *K. pneumoniae* SM9 growth, significant differences were observed when using both La-CFS vs. La-BS at 25% and 50% against the planktonic growth (*P* < 0.05), (Table S4D). In comparison, no significant differences were identified when 12.5% and 6.25% of CFS and BS were used.

Based on our findings, the CFS produced from lactobacilli showed an antibacterial and anti-biofilm potential higher than the biosurfactant against the tested CSOM-bacterial isolates.

## Discussion

The investigation of new antimicrobials is currently led by small research companies, institutes, and universities. Due to the low potential financial returns, such research has not received much investment or attention from pharmaceutical companies. The appearance of adverse side effects due to antibiotic use, such as microbial resistance and the re-occurrence of infection following treatment, highlights the urgent need for alternative antimicrobials and novel therapeutic methods. In this study, the antimicrobial potential of *L. acidophilus* and their metabolites, as naturally derived antimicrobials, was investigated against CSOM-associated pathogenic bacteria and their biofilm formation ability.

Our findings were in agreement or disagreement with several published studies. Bhuiya et al. [41] reported that an investigated *Pseudomonas* isolate was resistant to ceftazidime and aztreonam. Bacterial resistance to aztreonam and ceftazidime was also reported in a study by Al-Obadi [42], which disagrees with the results published by Hosseinzadeh et al. [43]. Regarding bacterial resistance to trimethoprim-sulfamethoxazole, our data on *Klebsiella* resistance to antibiotics agreed with Abbas & Jarallah [44] but disagreed with the observations of Mohsen et al. [45]. The recorded resistance of bacterial isolates to piperacillin was close to the findings of Kadhim [46]. Regarding the susceptibility of *P. mirabilis* to carbapenems groups, our results were close to Kadhim et al. [47] and were not comparable with the data reported by Pal et al. [48]. Levels of trimethoprim/sulfamethoxazole resistance are in agreement with the findings of Al-Bassam & Al-Kazaz [49]. Regarding *S. aureus*, Foster [50] reported that a *Staphylococcus* isolate was resistant to benzyl-penicillin due to the production of a beta-lactamase enzyme, which breaks down the beta-lactam ring, inactivating the antibiotic.

The differences in antibiotic susceptibility between our study and other published studies could be due to several reasons, such as the number of isolates, different working conditions, the health status of the patients, or the abuse of antibiotics for a long time. Bacterial resistance to antibiotics could be related to several factors: (i) the ability of bacteria to produce extended-spectrum beta-lactamase enzymes (ESβLs) [51]. These enzymes are encoded by genes carried on a plasmid or chromosome and are able to break down penicillins and cephalosporins and provide resistance to many antibiotics. (ii) modification of the target site and (iii) reducing the permeability of the outer wall, in addition to (iv) efflux systems that work on the excretion of antibiotics from within the cell [52].

Generally, the low MICs of La-CFS against the isolated pathogens could indicate its potent inhibitory effect. Several studies have highlighted the antimicrobial activity of lactobacilli CFS against bacterial pathogens. Avaiyarasi et al. [53] showed that the CFS of *L. acidophilus* inhibited the growth of *S. aureus*. Moreover, Ahn et al. [54] observed in their study that the investigated *Lactobacillus* strains possessed inhibitory activity against *P. aeruginosa* and *S. aureus*. Mohammed et al. [55] reported a remarkable inhibitory effect of CFS prepared from lactobacilli VB1 against tested pathogenic bacteria. Moreover, Muhsin & Hassan [56] erred to the importance of lactobacilli VB1 in reducing antibiotics–associated diarrhea when used in combination with levofloxacin. The mechanisms of antimicrobial activity of lactobacilli CSF include: (i) competitive exclusion of bacteria to adhere and compete for nutrients and adhesion receptors, (ii) coaggregation, the assembly of microbial communities into distinct, interlinked structures, (iii) production of antimicrobial compounds, such as lactic acid (lowers the pH), hydrogen peroxide (H_2_O_2_), biosurfactants, and bacteriocin like inhibitory substances (BLIS) [26].

The high prevalence of antibiotic-resistant bacteria is related to the misuse and overuse of conventional antibiotics, which reduces the efficiency of current treatments and leads to thousands of deaths [57]. Much attention has been paid to the use of alternative antibacterial therapies in fighting bacterial infections. Recent studies have shown that the combination of probiotics and antibiotics is more effective in eliminating pathogens than the use of antibiotics alone [58].

Our results were in agreement with several studies focused on the combination of probiotic CFS with commercial antibiotics. Aminnezhad et al. [59] reported synergistic interactions when different La-CFS were combined with ciprofloxacin against *P. aeruginosa* SM17. Similarly, Dasari et al. [60] also showed that the combination of *L*. *acidophilus* and ciprofloxacin had better effects than antibiotics alone. Furthermore, another study by Isayenko et al. [61] showed an increase in the diameter of zones of growth inhibition for *S. aureus* when the metabolic complexes of lactobacilli were combined with antibiotics.

The bactericidal activity of ciprofloxacin is related to its mechanism of action in blocking DNA replication through gyrase enzyme inhibition Dasari et al. [60]. Lactobacilli produce organic acids and H_2_O_2,_ which collapse the electrochemical proton gradient, altering cell membrane permeability and disrupting substrate transport systems [21]. Furthermore, lactic acid production increases environmental acidity and inhibits microbial growth [62].

Based on our data and the above-referenced published reports, we speculate that the antibacterial activity of La-CFS potentiates the bactericidal effect of ciprofloxacin when used in combination, leading to a higher inhibitory effect on bacterial pathogens [59]. Antimicrobial synergy has the following benefits: (i) expanding the antimicrobial spectrum; (ii) reducing the required dose of conventional antibiotics; (iii) neutralizing and reducing the toxicity of high concentrations of antibiotics; and (iv) preventing bacterial resistance [63].

Our study showed that *Lactobacillus acidophilus* VB1 has the potential to auto-aggregate and co-aggregate with the tested bacterial pathogens. Our findings were in agreement with the study of Tatsaporn & Kornkanok [64], who reported high auto-aggregation levels of LABs, 78–86% compared to *B. cereus*, *E. coli*, and *S. Typhimurium*, which were in the range of 61–65%.

The results of this work also showed that lactobacilli coaggregation with the isolated pathogenic bacteria gradually increased with time; the highest coaggregation was after 24 h of incubation. These data are similar to the data of Hojjati et al. [65], who reported a strong coaggregation (76%) between *Levilactobacillus*, formerly *Lactobacillus brevis* gp104, and *S. aureus* after 24 h of incubation. Our data are also in agreement with Al-Dulaimi et al. [36], who reported a high coaggregation potential after 24 h of incubation when the tested probiotics were mixed with the *Acinetobacter* isolates.

The beneficial effects of coaggregation of lactobacilli with pathogenic bacteria are associated with (i) their adherence to the mucosal surfaces of the host, (ii) enhancing the production of inhibitory substances, and (iii) competition with pathogens preventing colonization [66, 67]. Furthermore, the coaggregation of *Lactobacillus* species plays a role in inhibiting biofilm formation by different pathogenic bacteria through the production of proteolytic enzymes or a hole formed on the bacterial cell surface, which may lead to ATP efflux [68].

Biofilm formation is an important feature that aids pathogens in avoiding host immune responses, surviving at high concentrations of antibiotics, and causing chronic infections. Bacteria within a biofilm are 1000-fold more resistant to antibiotic treatment than planktonic cells [13]. If preliminary bacterial adhesion and subsequent biofilm formation can be prevented by CFS and/or BS produced by beneficial strains, chronic infections and the development of antimicrobial resistance can be lessened [69].

Probiotics, including lactic acid bacteria (LAB), especially lactobacilli, have been found to prevent or disperse pathogenic biofilms by attacking the bacterial membrane, resulting in a wrinkled membrane that may lead, eventually, to the inhibition of biofilm formation [69, 70]. This activity may be attributed to the ability of lactobacilli to interfere with harmful bacteria through competition for nutrients, coaggregation, and production of antimicrobials including bacteriocin, hydrogen peroxide, and organic acids [71].

Moreover, some studies have demonstrated that lactobacilli-derived CFS can integrate into the targeted biofilm and also compete with pathogens for adhesion sites, thereby blocking the first step of biofilm formation. Benmouna et al. [72] reported a significant decrease (66.29%) in *P. aeruginosa* biofilm treated with CFS of *Lactiplantibacillus plantarum*. Kaur et al. [73] noticed a reduction of 50–57% in the biofilm of *Vibrio cholerae, E. coli*, and *S. aureus* when the CFS of lactobacilli isolates was applied.

Regarding BS, its antibacterial mechanism is related to the disruption of the membrane structure by interacting with phospholipids and proteins of the bacterial cell membrane [74]. In addition, biosurfactants adhere to cell surfaces leading to the deterioration of the integrity of the cell membrane and the breakdown of the nutrition cycle [25].

Some studies illustrated the emulsification properties of BS as an anti-biofilm agent, which enhances the dispersal of formed biofilms or prevents the onset of pathogenic biofilm formation. Yan et al. [75] observed that BS can inhibit the adhesion and biofilm formation of *S. aureus*. In addition, Shaaban et al. [76] found that BS isolated from *L. acidophilus* inhibited the biofilm formation of *P. mirabilis* SM42.

Several new antimicrobial agents were registered recently, such as oritavancin and dalbavancin, two novel glycopeptides used to control acute skin and soft-tissue infections. New pharmacological compounds of interest have been approved during the same period [77]. Moreover, the antimicrobial combinations of ceftolozane and tazobactam, ceftazidime and avibactam, and meropenem and vaborbactam, in addition to cephalosporins with beta-lactamase inhibitors were applied to potentiate antibiotic activity against several multidrug-resistant bacteria. The data on antimicrobial combinations and their synergy in the preclinical research justifies further investment and investigations towards the development of novel alternative antimicrobials; however, attention must be paid to further clinical trials for these novel antimicrobials and their combination with antibiotics, which should be further evaluated by regulatory agencies [77]. It is worth mentioning that the outcomes of using those novel antimicrobial agents still require in-depth research and development to effectively control antibiotic resistance [78].

## Conclusion

Chronic suppurative otitis media, a polymicrobial and multidrug-resistant infection, requires urgent action to avoid its health-threatening complications. Ciprofloxacin is generally reported as the most effective antibiotic for controlling CSOM infections. However, microbial resistance to this antibiotic has been noticed and appears to be increasing with time. The high rates of antibiotic use in the treatment of CSOM may play a pivotal role in the development of antibiotic resistance, with dire consequences not only for the management of otitis media but nosocomial infections associated with the OM pathogens *P. aeruginosa*, *S. aureus*, and *Klebsiella pneumoniae* in particular, due to the alarming levels of multidrug-resistance among the ESKAPE group of pathogens. As such, alternative, effective, and safe therapeutic agents are urgently required, especially those with more limited potential for developing antimicrobial resistance. In this study, we concluded that using cell-free supernatants of *L. acidophilus* VB1 alone or in combination with ciprofloxacin will be, in the majority of cases, more effective than using ciprofloxacin alone against most CSOM-associated bacterial isolates and prevent their biofilm formation. However, further evaluations are required to investigate the potential of La-CFS to eradicate the pre-formed biofilm.

## Electronic Supplementary Material

Below is the link to the electronic supplementary material.

Supplementary Material 1
