# Supplementary material for: Lactobacillus acidophilus VB1 co-aggregates and inhibits biofilm formation of chronic otitis media-associated pathogens
Source: Braz J Microbiol. 2024 May 24;55(3):2581–92. doi: 10.1007/s42770-024-01363-5 (PMC11405553; doi:10.1007/s42770-024-01363-5)
Supplement: Supplementary file 1 — Supplementary Material 1 [file 42770_2024_1363_MOESM1_ESM.docx]

**Supplementary Tables**

**Table S1 The source, and the designation of the bacterial isolates.**

| **Bacterial species** | **Bacterial sources** | **Strain designation** |
| --- | --- | --- |
| *Lactobacillus acidophilus* | Vitalactic B | ***L. acidophilus* VB1** |
| *Pseudomonas aeruginosa* | Laboratory collection | ***P. aeruginosa* SM17** |
| *Staphylococcus aureus* | Laboratory collection | ***S. aureus* SM23** |
| *Klebsiella pneumonia* | Laboratory collection | ***K. pneumoniae* SM9** |
| *Proteus mirabilis* | Laboratory collection | ***P. mirabilis* SM42** |

**Table S2 Antibacterial activity of two-fold dilutions of ciprofloxacin against bacterial isolates. Results are expressed as mean bacterial growth inhibition ± SD (μg** **ml^-1^) to three independent experiments.**

| **Ciprofloxacin (μg ml^-1^)** | **Mean ± SE**  **(bacterial growth inhibition %)** | ***P*** |
| --- | --- | --- |
| **0** | 0.00 **±** 0.00 | Zero vs. **0.95**  <0.000  Zero vs. **1.9**  <0.000  Zero vs. **3.9** <0.000  Zero vs. **7.8**  <0.000  Zero vs. **15.6** <0.000  Zero vs. **31.3** <0.000 |
| **0.95** | 71.00 **±** 14.34 |  |
| **1.9** | 94.61 **±** 4.44 |  |
| **3.9** | 98.87 **±** 0.42 |  |
| **7.8** | 99.75 **±** 0.25 |  |
| **15.6** | 100.00 **±** 0.00 |  |
| **31.3** | 100.00 **±** 0.00 |  |

**Table S3 Antibacterial activity of two-fold dilutions of La-CFS against bacterial isolates. Results are expressed as mean bacterial growth inhibition ± SD (%) to three independent experiments.**

| **La-CFS Concentration (%)** | **Mean ± SE**  **(bacterial growth inhibition %)** | ***P*** |
| --- | --- | --- |
| **0** | 0.00 **±** 0.00 | Zero vs. **6.25** = 0.698  Zero vs. **12.5** = 0.026  Zero vs. **25** = <0.000  Zero vs. **50** = <0.000 |
| **6.25** | 3.33 **±** 1.12 |  |
| **12.5** | 19.46 **±** 5.75 |  |
| **25** | 67.90 **±** 12.14 |  |
| **50** | 98.32 **±** 0.08 |  |

**Table S4 Effect of CFS and BS of *L. acidophilus*** VB1 **on biofilm and planktonic growth of the bacterial species. Results expressed as mean MBIC50±SD (%) to three independent experiments.**

**Table S4A Effect of CFS and BS of *L. acidophilus*** VB1 **on *P. aeruginosa*** SM17**.**

| ***P. aeruginosa*** SM17 **biofilm inhibition %** | | | |
| --- | --- | --- | --- |
| **Concentration (%)** | **La-CFS**  **Mean ± SE** | La-BS  Mean ± SE | *P* |
| **50** | 100.00 ± 0.00 | 75.70±1.18 | 0.002 |
| **25** | 68.89± 6.01 | 27.47± 0.00 | 0.020 |
| **12.5** | 59.62± 4.89 | 14.28± 4.39 | 0.020 |
| **6.25** | 41.05± 2.61 | 3.29± 3.29 | 0.012 |
| ***P. aeruginosa*** SM17 **growth inhibition %** | | | |
| **Concentration (%)** | **La-CFS**  **Mean ± SE** | **La-BS**  **Mean ± SE** | ***P*** |
| **50** | 3.50± 1.15 | 15.35± 1.12 | 0.018 |
| **25** | 99.49± 4.19 | 97.24± 3.16 | 0.710 |
| **12.5** | 96.37± 1.80 | 101.87± 1.30 | 0.132 |
| **6.25** | 101.30± 0.73 | 98.62±2.49 | 0.411 |

**Table S4B Effect of CFS and BS of *L. acidophilus*** VB1 **on *S. aureus*** SM23**.**

| ***S. aureus*** SM23 **biofilm inhibition %** | | | |
| --- | --- | --- | --- |
| **Concentration (%)** | **La-CFS**  **Mean ± SE** | **La-BS**  **Mean ± SE** | ***P*** |
| **50** | 100.00±0.00 | 44.21±0.59 | <0.000 |
| **25** | 38.87±1.18 | 29.37±5.34 | 0.225 |
| **12.5** | 30.61±4.76 | 11.86±0.89 | 0.061 |
| **6.25** | 5.77±2.22 | 4.74±2.67 | 0.797 |
| ***S. aureus*** SM23 **growth inhibition %** | | | |
| **Concentration (%)** | **La-CFS**  **Mean ± SE** | **La-BS**  **Mean ± SE** | ***P*** |
| **50** | 4.00±1.00 | 37.60±2.31 | 0.006 |
| **25** | 49.85±4.92 | 68.34±5.32 | 0.126 |
| **12.5** | 63.76±1.44 | 77.10±0.63 | 0.014 |
| **6.25** | 65.65±0.34 | 77.59±0.84 | 0.006 |

**Table S4C Effect of CFS and BS of *L. acidophilus*** VB1 **on *P. mirabilis*** SM42**.**

| ***P. mirabilis*** SM42 **biofilm inhibition %** | | | |
| --- | --- | --- | --- |
| **Concentration (%)** | **La-CFS**  **Mean ± SE** | **La-BS**  **Mean ± SE** | ***P*** |
| **50** | 100 ± 0.00 | 77.97 ± 3.07 | 0.019 |
| **25** | 76.83±2.32 | 64.67±3.21 | 0.092 |
| **12.5** | 65.12±1.93 | 48.16±1.37 | 0.019 |
| **6.25** | 55.40±1.73 | 45.41±1.37 | 0.046 |
| ***P. mirabilis*** SM42 **growth inhibition %** | | | |
| **Concentration (%)** | **La-CFS**  **Mean ± SE** | **La-BS**  **Mean ± SE** | ***P*** |
| **50** | 3.50±1.15 | 38.56±5.23 | 0.023 |
| **25** | 21.77±4.05 | 67.45±6.02 | 0.024 |
| **12.5** | 68.91±5.55 | 78.77±2.85 | 0.256 |
| **6.25** | 101.30± 0.73 | 98.62±2.49 | 0.411 |

**Table S4D Effect of CFS and BS of *L. acidophilus*** VB1 **on *K. pneumoniae*** SM9.

| ***K. pneumonia*** SM9 **biofilm inhibition %** | | | |
| --- | --- | --- | --- |
| **Concentration (%)** | **La-CFS**  **Mean ± SE** | **La-BS**  **Mean ± SE** | ***P*** |
| **50** | 100.00 **±** 0.00 | 57.48 **±** 4.18 | 0.010 |
| **25** | 52.97± 3.01 | 55.66 **±** 4.42 | 0.658 |
| **12.5** | 36.36 **±** 7.15 | 29.94 **±** 5.88 | 0.570 |
| **6.25** | 20.39 **±** 2.48 | 17.29 **±** 3.55 | 0.549 |
| ***K. pneumonia*** SM9 **growth inhibition %** | | | |
| **Concentration (%)** | **CFS**  **Mean ± SE** | **BS**  **Mean ± SE** | ***P*** |
| **50** | 3.50 **±** 1.50 | 93.93 **±** 1.09 | <0.000 |
| **25** | 83.26± 1.39 | 96.11 **±** 1.54 | 0.025 |
| **12.5** | 98.02 **±** 0.26 | 98.90**±** 1.39 | 0.599 |
| **6.25** | 101.04 **±** 0.81 | 99.05 **±** 1.39 | 0.344 |
